# Hypoxia and aging

**DOI:** 10.1038/s12276-019-0233-3

**Published:** 2019-06-20

**Authors:** Eui-Ju Yeo

**Affiliations:** Department of Biochemistry, College of Medicine, Gachon University, Incheon, 21999 Republic of Korea

**Keywords:** Senescence, Experimental models of disease

## Abstract

Eukaryotic cells require sufficient oxygen (O_2_) for biological activity and survival. When the oxygen demand exceeds its supply, the oxygen levels in local tissues or the whole body decrease (termed hypoxia), leading to a metabolic crisis, threatening physiological functions and viability. Therefore, eukaryotes have developed an efficient and rapid oxygen sensing system: hypoxia-inducible factors (HIFs). The hypoxic responses are controlled by HIFs, which induce the expression of several adaptive genes to increase the oxygen supply and support anaerobic ATP generation in eukaryotic cells. Hypoxia also contributes to a functional decline during the aging process. In this review, we focus on the molecular mechanisms regulating HIF-1α and aging-associated signaling proteins, such as sirtuins, AMP-activated protein kinase, mechanistic target of rapamycin complex 1, UNC-51-like kinase 1, and nuclear factor κB, and their roles in aging and aging-related diseases. In addition, the effects of prenatal hypoxia and obstructive sleep apnea (OSA)-induced intermittent hypoxia have been reviewed due to their involvement in the progression and severity of many diseases, including cancer and other aging-related diseases. The pathophysiological consequences and clinical manifestations of prenatal hypoxia and OSA-induced chronic intermittent hypoxia are discussed in detail.

## Aging: Low oxygen levels promote aging-associated diseases

Deficient oxygen supplies to the body’s tissues and a reduced ability to deal with the cellular stress of low oxygen availability both contribute to the ageing process. Eui-Ju Yeo from Gachon University in Incheon, South Korea, reviews the links between insufficient oxygen, a condition known as hypoxia, and the functional decline associated with getting old. Yeo highlights the role that the oxygen-sensing proteins known as hypoxia-inducible factors (HIFs) play in facilitating oxygen supply and regulating energy production under limited oxygen conditions. HIFs also interact with a series of signaling proteins that, when abnormally activated, contribute to many aging-associated diseases. Yeo singles out two hypoxia-related health problems, prenatal hypoxia during early brain development and obstructive sleep apnea, and discusses how they can accelerate aging in various ways.

## Introduction

Oxygen (O_2_) plays critical roles in aerobic respiration and metabolism as the final electron acceptor of the mitochondrial electron transport chain, which is responsible for generating the majority of ATP within an eukaryotic cell^1^. Therefore, an adequate concentration of oxygen is required by eukaryotic cells to maintain a variety of biological activities and ensure survival. When the oxygen demand exceeds its supply, oxygen levels in the whole body or local tissues decrease (termed hypoxia), leading to a metabolic crisis, threatening physiological functions and viability. Due to the critical roles of oxygen in respiration, metabolism, and survival, eukaryotes have developed an efficient and rapid oxygen sensing system^1^.

A change in the oxygen concentration in the surrounding atmosphere is detected by central and arterial chemoreceptors^2^. Central chemoreceptors are present in the medulla of the brainstem near the respiratory centers. Arterial chemoreceptors are present in the aortic body and carotid body. The activation of arterial chemoreceptors stimulates the neurotransmitter release pathway^3^ and modulates the activity of a neutral endopeptidase, neprilysin (NEP), which modifies the cellular response to hypoxia by hydrolyzing substance P^4^. Oxygen sensing is also controlled by pulmonary neuroendocrine cells in neuroepithelial bodies, exerting chemosensitivity, which is important for oxygen sensing in early developmental stages of life^5^. These stimulated chemoreceptors increase sympathetic neural activity (SNA) and the arterial pulmonary and systemic blood supply to obtain more oxygen^6^. On the other hand, the expression of several adaptive genes is induced to increase the oxygen supply and support anaerobic ATP generation in eukaryotic cells. These hypoxic responses are controlled by hypoxia-inducible factors (HIFs)^7,8^. Over the past two decades, various biochemical and genetic studies have expanded our knowledge of hypoxia to the cellular and molecular levels. Therefore, the HIF pathway is briefly summarized in the first section “Hypoxia and the HIF pathway”.

In addition, hypoxia potentially contributes to functional decline during the aging process. HIF pathways cross-talk with the sirtuins, AMP-activated protein kinase (AMPK), mechanistic target of rapamycin complex 1 (mTORC1), UNC-51-like kinase 1 (ULK1), and nuclear factor κB (NFκB) pathways in hypoxia and aging^9–12^. The putative molecular mechanisms underlying the effects of hypoxia, including HIF-1α, AMPK, sirtuins, mTORC1, ULK1, and NFκB, are discussed in the section “HIF-1α and aging”.

Traditionally, physiological research has focused on the effects of acute and chronic sustained hypoxia and the human adaptive response to high altitude. However, in recent years, the effects of obstructive sleep apnea (OSA)-induced intermittent hypoxia and prenatal hypoxia have garnered interest due to the accelerated progression and severity of many diseases, including cardiovascular and metabolic diseases, neurological disorders (including depression and neurodegenerative diseases), cancer, and aging. Therefore, the pathophysiological consequences and clinical manifestations of OSA-induced chronic intermittent hypoxia and prenatal hypoxia are discussed here in detail.

## Hypoxia and the HIF pathway

### Regulation of HIF-1α expression and activity during hypoxia

Because of its involvement in a diverse range of disease states, the mechanism by which cells sense hypoxia and transduce a signal to the HIF pathway has been intensively investigated by many researchers. Members of the human HIF family (HIF-1, HIF–2, and HIF–3) are heterodimeric transcription factors, each of which is composed of an α and a β subunit (also termed as aryl hydrocarbon receptor nuclear translocator; ARNT)^13,14^. Cellular and developmental responses to hypoxia are mainly mediated by HIF-1α, which is encoded by the *HIF1A* gene^13,15^. Three HIF-1α isoforms have been identified (HIF-1α, HIF-2α, HIF-3α), which are generated by alternative splicing. Among the three isoforms, isoform 1 has been extensively studied and characterized, both structurally and functionally^16^. The dysregulation and overexpression of *HIF1A* induced by genetic alternations and hypoxia have been implicated in a number of pathophysiologies, including ischemia and cancer^17^. HIF-1α contains a basic helix-loop-helix domain, two distinct PAS (PER-ARNT-SIM) domains, a nuclear localization signal motif, two (carboxy-terminal and amino-terminal) transactivating domains (CTAD and NTAD, respectively), and an inhibitory domain that represses the transcriptional activities of CTAD and NTAD^18^. In contrast to HIF-1α, which is upregulated under hypoxia, HIF-1β is expressed constitutively.

HIF-1 responds to systemic oxygen levels by interacting with hypoxia responsive elements (HRE) located in the promoter or enhancer regions of hypoxia-responsive genes to induce transcription^19–24^. HIF-1 induces the transcription of more than 100 genes^25,26^ involved in various biological processes, such as angiogenesis, erythropoiesis, anaerobic glycolytic metabolism, pH regulation, cell proliferation and survival, inflammation and immunity, and cancer metastasis (Table 1). HIF-1 induces the expression of several pro-angiogenic factors, including vascular endothelial growth factor (VEGF), which promotes the formation of new blood vessels and oxygen delivery to hypoxic regions^17,24,27,28^. In addition, HIF-1 increases oxygen transport by enhancing erythropoiesis through the upregulation of erythropoietin (EPO). HIF-1 also stimulates the uptake and anaerobic glycolysis of glucose by increasing the expression of glucose transporters, such as GLUT1 and GLUT3, and several glycolytic enzymes, including hexokinases. HIF-1 activation increases the conversion of pyruvate to lactate by upregulating pyruvate kinase and lactate dehydrogenase. HIF-1 promotes lactic acid efflux by monocarboxylate transporter and the conversion of CO_2_ to carbonic acid (H_2_CO_3_) by carbonic anhydrase to counteract intracellular acidosis. Furthermore, HIF-1 affects cell proliferation and survival by modulating the expression of C-MYC, insulin-like growth factor 2 and other components of the cell cycle and death pathways. HIF-1 also induces inflammation and immunity by upregulating tumor necrosis factor α (TNFα) and cancer metastasis by upregulating fibronectin 1. The functions of HIF-1 itself and HIF-1 target genes have been effectively examined using siRNA knockdown-based validation^29^.

**Table 1:** Representative HIF-1 target gene products

Functional category	Symbol	Full name
Angiogenesis	*VEGF*	Vascular endothelial growth factor
	*NOS*	Nitric oxide synthase
	*LEP*	Leptin
	*LRP1*	LDL-receptor-related protein 1
	*ADM*	Adrenomedullin
	*TGF-β3*	Transforming growth factor-β3
Erythropoiesis	*EPO*	Erythropoietin
Anaerobic glycolytic metabolism	*GLUT1/3*	Glucose transporter 1 and 3
	*HK1/2*	Hexokinase 1 and 2
	*PFK*	6-phosphofructo-2-kinase
	*FBPase*	Fructose-2,6-bisphosphatase
	*PKM*	Pyruvate kinase muscle isozymes (M1/M2)
	*PDK1*	Pyruvate dehydrogenase kinase 1
	*LDHA*	Lactate dehydrogenase A
pH regulation	*MCT4*	Monocarboxylate transporter 4
	*CA9*	Membrane-associated carbonic anhydrase IX
Cell proliferation and survival	*C-MYC*	Myelocytomatosis virus oncogene cellular homolog
	*IGF2*	Insulin-like growth factor 2
	*IGF-BP 1/2/3*	IGF-binding protein 1/2/3
	*ID2*	DNA-binding protein inhibitor
	*ADM*	Adrenomedullin
	*iNOS*	Inducible nitric oxide synthase
	*TGF-α*	Transforming growth factor α
	*VEGF*	Vascular endothelial growth factor
Inflammation and immunity	*TNF-α*	Tumor necrosis factor α
	*Rags*	Recombination activating gene products
	*TASK-2*	Potassium channels in B cells
Cancer metastasis	*FN1*	Fibronectin 1
	*LOXL2*	Lysyl oxidase-like 2
	*uPAR*	Urokinase plasminogen activator receptor

A variety of factors are implicated in the mechanism regulating HIF-1α expression and activity^25^. HIF-1α expression is controlled at the levels of transcription and translation. In addition, several factors regulate the stability and activity of HIF-1α at the post-translational level in an oxygen-dependent or oxygen-independent manner. The key factors that regulate its abundance and activity are summarized in Table 2.

**Table 2:** Factors that regulate the abundance and activity of HIF-1α

Regulation stage	Modulators	Change in the level of HIF-1α
Transcription	NFκB	**↑**
	STAT3	**↑**
	p300/CBP	**↑**
	ROS (by activating NFκB)	**↑**
Translation	IRES elements	**↕**
	RNA-binding proteins (PTB and HuR)	**↑**
	PI3K and MAPK signaling pathways	**↑**
	Calcium signaling	**↕**
	miRNAs	**↕**
Post-translational degradation	PHD, pVHL, OS-9 and SSAT2 (oxygen-dependent)	**↓**
	ARD-1 (arrest-defective-1: oxygen-dependent acetylation of HIF-1α)	**↓**
	Mdm2-p53 (ubiquitination and proteasomal degradation, oxygen-independent)	**↓**
	EPF UCP (degrades pVHL, oxygen-dependent)	**↑**
	VDU2 (de-ubiquitinates HIF-1α, oxygen-dependent)	**↑**
	SUMOylation by RSUME (oxygen-dependent)	**↑**
	DeSUMOylation by SENP1 (oxygen-dependent)	**↑**
	Calcineurin A (Ca^2+^-dependent dephosphorylation of RACK1, oxygen-independent)	**↑**
	Hsp90 (oxygen-independent)	**↑**
	RACK1 and SSAT1 (oxygen-independent)	**↓**
	GSK3β and FoxO4 (regulated by PI3K and PTEN, oxygen-independent)	**↓**

*↑ indicates an increase, ↓ indicates a decrease, and ↕ indicates either an increase or decrease in HIF-1α levels

Although the *HIF1A* gene is constitutively expressed at low levels under normoxic conditions, it is often significantly upregulated in response to hypoxia^30–35^. The transcriptional modulators of HIF-1α expression include transcription factors, such as nuclear factor kappa B (NFκB) and signal transducer and activator of transcription 3 (STAT3), and coactivators, such as p300/CREB binding protein (CBP)^36,37^. The expression of the *HIF1A* gene may also be upregulated by reactive oxygen species (ROS) through the redox-sensitive transcription factor, NF-κB^38^. Vanillin possesses anti-metastatic and anti-cancer activities and inhibits STAT3-mediated HIF-1α mRNA expression in human malignant melanoma cells^39^. Similarly, YC-1, 3-(5-hydroxymethy-2-furyl)-1-benzylindazole, functions as a potential anticancer drug targeting HIF-1^40^, presumably by inhibiting NFκB-mediated and STAT3-mediated transcription of the *HIF1A* gene.

**Fig. 1:**
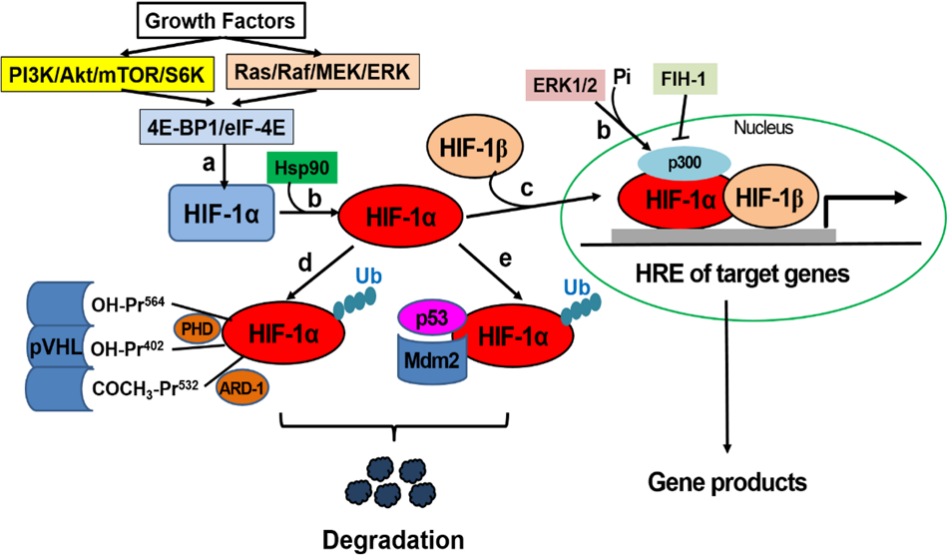
Regulation of the HIF-1α pathway. **a** Growth factor-induced HIF-1α protein synthesis pathways. **b** HIF-1α transactivation through an interaction with Hsp90 or p300 that is phosphorylated by ERK1/2. **c** Heterodimerization of HIF-1α and HIF-1β. **d** VHL-related degradation pathways. **e** Mdm2/p53-mediated ubiquitination and proteasomal degradation pathway

Although general protein translation decreases during hypoxia, HIF-1α continues to be translated. This event has been confirmed by several studies using translation and proteasomal inhibitors, and reporter assays with the 5’ UTR of *HIF1A* gene. However, the exact mechanism by which *HIF1A* is selectively translated during hypoxia remains unclear. One postulated mechanism includes the internal ribosome-entry-site (IRES) element within the 5’ UTR of the HIF-1α mRNA^41^. The IRES element forms special secondary or tertiary structures, enabling ribosome binding in the absence of the eIF4F cap-binding complex. RNA-binding proteins, such as polypyrimidine tract-binding protein (PTB) and HuR, also bind the 3’ UTR and 5’ UTR of the HIF-1α mRNA, respectively, promoting HIF-1α translation^42^. HIF-1α protein synthesis is also regulated indirectly by growth factor-induced signal transduction (Fig. 1a), including the phosphatidylinositol 3 kinase (PI3K), mitogen-activated protein kinase (MAPK), and calcium signaling pathways^25^. However, the effects of translational modulators are still controversial and remain to be clarified.

HIF-1α activity is regulated by proteasomal degradation induced by specific post-translational modifications: hydroxylation, acetylation, SUMOylation, and phosphorylation^24,25^. Under normoxic conditions, the von Hippel-Lindau tumor suppressor (pVHL)-mediated ubiquitin proteasome pathway rapidly degrades HIF-1α; however, under hypoxic conditions, HIF-1α protein degradation is prevented and the activated α subunit forms a heterodimer with the β subunit to function as a transcription factor (Fig. 1c). The phosphorylation of a coactivator, p300/CBP, by ERK1/2 promotes HIF-1α-dependent transactivation of target genes (Fig. 1b). The oxygen sensitivity of the HIF pathway is conferred by prolyl hydroxylase (EGLN 1-3, also known as PHD 1-3) and HIF prolyl hydroxylase (HPH)^25,43^. These hydroxylases are involved in the hydroxylation of HIF-1α proline residues (Pro402 and Pro564) using oxygen as a substrate (Fig. 1d). In addition to oxygen, this reaction requires 2-oxoglutarate as a cosubstrate and ascorbate and iron as cofactors^22^. Because PHDs and HPH use iron as a cofactor, iron chelators, such as desferrioxamine (DFO), effectively inhibit PHDs and stabilize HIF-1α^44^. Similarly, cobalt chloride (CoCl_2_) is an well-known hypoxia mimetic agent that blocks the catalytic activity of PHDs by competing with the bivalent ion^45^, leading to HIF-1α stabilization^46,47^.

The pVHL protein associates with elongins B and C, cullin 2, and ring-box 1 (Rbx1) to form an E3 ubiquitin-ligase complex, p**V**HL-**E**longin B/C-**C**ullin 2 (VEC), and subsequently ubiquinates the α subunit of HIF-1. The pVHL protein functions as the substrate-docking interface that specifically recognizes prolyl-hydroxylated HIF-1α^48,49^. Ubiquitin-tagged HIF-α proteins are subsequently degraded by the common 26S proteasome (Fig. 1d)^23,50^. This hydroxylation only occurs under normoxic conditions, i.e., in the presence of oxygen. Therefore, during hypoxia, HIF-1α escapes degradation and dimerizes with the common and constitutively stable HIF-1β protein (Fig. 1c). Acetylation of HIF-1α at Lys532 by arrest-defective-1 (ARD-1) also facilitates its recognition by pVHL and subsequent degradation under normoxic conditions (Fig. 1d)^51^. Other regulators of HIF-1α include osteosarcoma-9 (OS-9) and spermidine/spermine-N^1^-acetyltransferase 2 (SSAT2). OS-9 forms a ternary complex with PHDs and HIF-1α, promoting proline hydroxylation and subsequent pVHL-dependent HIF-1α degradation. SSAT2 binds to pVHL and elongin C, thereby stimulating HIF-1α ubiquitination and degradation. On the other hand, Mdm2/p53-mediated ubiquitination and proteasomal degradation pathways are involved in regulating HIF-1α levels in an oxygen-independent manner (Fig. 1e).

HIF-1α activity is controlled via the degradation of pVHL by a member of the E2 enzyme family, endemic pemphigus foliaceus ubiquitin carrier protein (E2-EPF UCP), and de-ubiquitination of HIF-1α by the pVHL-interacting de-ubiquitylating enzyme (VDU2, USP20). However, the role of hypoxia-induced SUMOylation of HIF-1α remains unclear^25^. In general, hypoxia results in HIF-1α SUMOylation, which facilitates the recognition of HIF-1α by the pVHL E3 ligase complex, leading to HIF-1α degradation. Sentrin specific peptidase 1 (SENP1), a nuclear SUMO protease, reverses this modification, resulting in its stabilization during hypoxia^52^. However, the RWD-containing SUMOylation enhancer (RSUME)-induced SUMOylation of HIF-1α results in increased HIF-1α protein levels and transactivation^53^.

The stability of the HIF-1α protein is also regulated by oxygen-independent modulators, including a calcium-dependent and calmodulin-dependent serine/threonine protein phosphatase (calcineurin A), a heat-shock protein (Hsp90), receptor of activated protein kinase C (RACK1) and GSK3β. Calcineurin A dephosphorylates RACK1, thus inhibiting RACK1 dimerization and RACK1-dependent HIF-1α degradation^54^. Hsp90 binds to HIF-1α, stabilizing the protein. Hence, Hsp90 inhibitors, such as 17-(allylamino)-17-demethoxygeldanamycin (17AAG), cause oxygen-independent HIF-1α degradation. In contrast, the RACK1 homodimer binds to HIF-1α upon Hsp90 inhibition (by 17AAG) and recruits components of the E3 ligase complex. SSAT1 (inactive) stabilizes the RACK1-HIF-1α interaction, leading to HIF-1α ubiquitination and degradation^55^. GSK3β phosphorylates HIF-1α, leading to its ubiquitination and degradation. Hypoxia might inhibit the PI3K/Akt pathway, causing the activation of GSK3β and FoxO4 to subsequently decrease HIF-1α levels^56^. The PI3K pathway is activated by growth factors and inhibited by phosphatase and tensin homolog (PTEN) or serum depletion.

## Hypoxia and cancer

Hypoxia plays important roles in cancer initiation, progression, and metastasis. Hypoxia is mainly observed in the tumor environment as the blood supply soon becomes inadequate for the rapidly growing tumor cells. Hypoxia activates HIF-1 to promote carcinogenesis and tumor growth by regulating the expression of genes involved in angiogenesis, glycolytic metabolism, and other biological mechanisms (Table 1). Once hydroxylated under normoxic conditions, HIF-1 binds to the tumor suppressor protein, pVHL, which induces its proteasomal degradation. However, during hypoxia, the HIF-1 heterodimer undergoes stabilization and nuclear translocation, leading to the activation of several oncogenes. Hypoxic regulation of oncogenes by pVHL provides insights into cancer pathogenesis^57^. The role of pVHL in regulating HIF-1α activity is evident in von Hippel Lindau disease. Inactivation of the *VHL* gene induces the development of highly vascularized tumors in various organs, such as the kidney and central nervous system^58^. Recent evidence also support the hypothesis that hypoxia and hypoxia-related pathways play important roles in the development and progression of a subtype of renal cell carcinomas (clear cell RCC: 70–75%), which is associated with the loss of pVHL function^59^, high levels of HIF-1, and thus deregulation of hypoxia pathways^60–63^. Interestingly, the two HIF-α proteins (HIF-1α and HIF-2α) exert opposite effects on clear cell RCC, with HIF-1α functioning as a tumor suppressor and HIF-2α acting as an oncogene^64^. Moreover, because UCP ubiquitinates pVHL, leading to its degradation^65^, UCP overexpression causes pVHL degradation, resulting in HIF-1α accumulation and increased tumor growth and metastasis. Conversely, UCP knockdown increases pVHL levels, causing a decrease in HIF-1α levels and inhibiting tumor growth^65^. In addition to the pVHL, HIF-1α and UCP proteins, a variety of modulators of HIF-1α (Table 2) have been implicated in carcinogenesis and cancer metastasis, as recently reviewed by Aldo and Elisabetta^66^.

## Differences in the cellular responses to sustained hypoxia and intermittent hypoxia

Interestingly, the inhibition of mitochondrial function reverses the hypoxia-induced HIF pathway. These observations prompted researchers to propose a role for the mitochondria in cellular oxygen sensing. Two models have been proposed to describe the role of mitochondria in oxygen sensing, implicating ROS and oxygen redistribution, respectively^1^. These two models may complement each other and facilitate the rapid and dynamic activation of the HIF pathway in response to hypoxia.

**Fig. 2:**
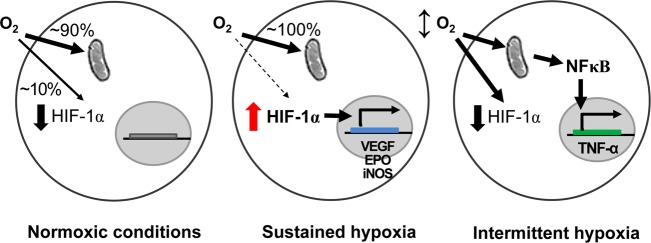
Hypoxia and HIF-1α stabilization. Under normoxic conditions, mitochondrial respiration consumes more than 90% of oxygen in humans. The remaining cytosolic oxygen (approximately 10%) is sufficient for HIF-1α degradation. During sustained hypoxia, the mitochondria use up almost all the oxygen and the free cytosolic oxygen is exhausted, causing HIF-1α stabilization. Activated HIF-1α induces the transcription of many genes, such as VEGF, EPO, and iNOS. However, the response to intermittent hypoxia differs from the response to sustained hypoxia. Intermittent hypoxia may not remove a sufficient amount of cytosolic free oxygen to allow HIF-1α stabilization; instead, intermittent hypoxia activates NFκB probably through mitochondrial stress, resulting in the production of TNF-α, an inflammatory mediator

Under normoxic conditions, mitochondrial respiration consumes greater than 90% of the oxygen in humans. The remaining oxygen (~10%) is utilized for HIF-1α degradation. In response to sustained hypoxia, the mitochondria consume almost all the oxygen and remove free cytosolic oxygen, causing the rapid stabilization of HIF-1α. Activated HIF-1α leads to the increased transcription of a number of genes, such as VEGF, EPO, and inducible nitric oxide synthase (iNOS). These factors participate in the adaptive response to hypoxia by increasing tissue perfusion and oxygenation, and hence aid in the recovery from the initial hypoxic insults. However, the molecular responses activated by intermittent hypoxia differ from those activated by sustained hypoxia. In intermittent hypoxia, a free oxygen deficit is not sufficiently established to allow HIF-1α stabilization; instead, intermittent hypoxia results in the activation of NFκB, possibly through mitochondrial stress, triggering the production of inflammatory mediators, such as TNF-α (Fig. 2)^67^. The level of HIF-1α is also regulated transcriptionally in an NFκB-dependent manner^36^. Intermittent hypoxia may cause a delayed increase in HIF-1α, resulting in the activation of NFκB-driven inflammation, possibly as a result of oxidative stress^67–70^.

## HIF-1α and aging

### Cross-talk between the HIF-1α and SIRT1 pathways during aging

Aging is a degenerative process leading to cellular dysfunction, tissue failure, and a deterioration of bodily functions. Two well-known theories, the “free radical theory of aging” and “mitochondrial theory of aging” (Bereiter-Hahn, 2014), claim that mitochondria-induced oxidative damage results in detrimental effects on mitochondrial function, leading to ROS production and inflicting further injury^71,72^.

**Fig. 3:**
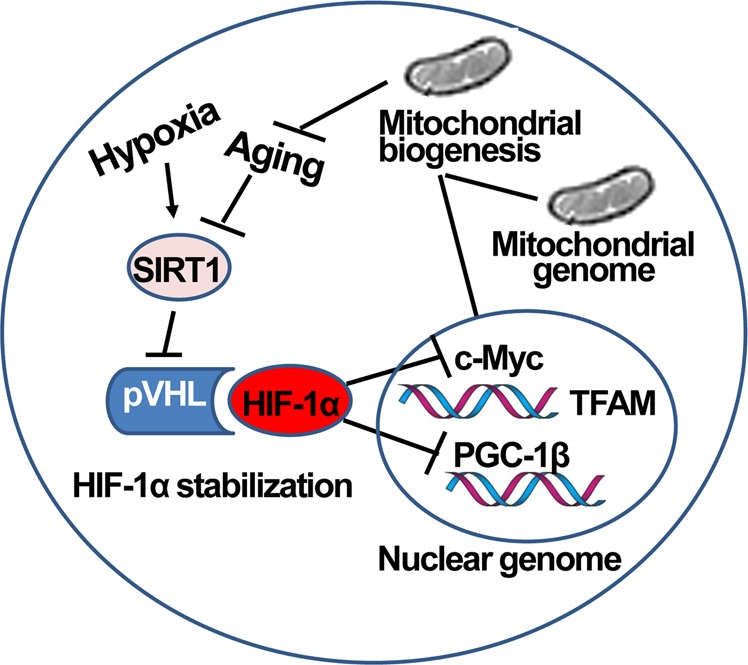
Regulation of mitochondrial biogenesis and aging by SIRT1 and HIF-1α. During the aging process, the activity of SIRT1 in the nucleus is reduced, which decreases pVHL levels and subsequently stabilizes HIF-1α. Activated HIF-1α reduces c-Myc activity and subsequently reduces the transcription of TFAM, which is required for replication, transcription, and maintenance of mitochondrial biogenesis. PGC-1β activity is also inhibited upon its interaction with HIF-1α, resulting in the downregulation of mitochondrial genes

During the aging process, HIF-1α induces a deficit in mitochondrial biogenesis (Fig. 3), which impairs energy-dependent cellular processes, including cell and tissue repair^73^. The subsequent accumulation of ROS, oxidation of lipids and proteins, and mutations in mitochondrial DNA accelerate the aging process by inducing a deterioration of cellular energetics, the cellular redox state, calcium homeostasis, and cell signaling^73^. Therefore, the mechanisms regulating HIF-1α have been implicated in the prevention of premature cellular senescence and the pathogenesis of many aging-related chronic diseases.

SIRT1 is a member of the sirtuin family, sharing homology with *Saccharomyces cerevisiae* silent information regulator 2 (Sir2), a highly conserved protein with dual enzymatic activities: nicotinamide adenine dinucleotide (NAD^+^)-dependent deacetylases and ADP-ribosyltransferase activities. Although this family is composed of seven members, SIRT1−7^74^, SIRT1 is the most extensively studied member due to its involvement in the mechanisms regulating various important biological and pathological processes, including apoptosis, inflammation, and senescence^75^, which are associated with aging-related diseases. Studies of the transcriptional regulation of the SIRT1 gene in metabolism have revealed critical roles for peroxisome proliferator-activated receptor (PPAR)-β/δ and the canonical Sp1 binding site in the 5’ SIRT1 promotor^76^. Sp1 overexpression significantly enhances SIRT1 promoter activity by binding to the Sp1 element, as confirmed by EMSA and ChIP assays and experiments using a specific Sp1 antagonist, mithramycin. Two miRNAs, miR-34a and miR-93, are known to be upregulated; however, the expression of their candidate targets, Sp1 and SIRT1, are reduced during aging^77^. A reduction in Sp1 activity might be responsible for the decrease in SIRT1 transcription, and subsequent suppression of angiotensin II-induced cardiac hypertrophy by flavonol (−)-epicatechin^78^.

Interestingly, the level of SIRT1 decreases in both transcriptional and posttranscriptional stages during aging, which attenuates mitochondrial biogenesis and causes aging-related diseases. Aging-associated reductions in the nuclear energy state and NAD^+^ levels decrease SIRT1 activity, resulting in diminished pVHL levels and the stabilization of HIF-1α (Fig. 3). Conversely, overexpression of SIRT1 promotes mitochondrial biogenesis by deacetylation, resulting in the activation of HIF-1α^79^ (Fig. 3) and PPARγ co-activator 1α (PGC-1α)^80^. The upregulation of SIRT1 has been implicated in the prevention of premature cellular senescence and the pathogenesis of many aging-related chronic diseases^81–83^. SIRT1 is the factor that is activated by caloric restriction and natural polyphenolic compounds, such as resveratrol, which is responsible for increasing longevity. Therefore, overexpression of SIRT1 results in delayed aging phenotypes and an extension of the lifespan, whereas inhibition of SIRT1 abrogates the extension of the lifespan in mice^84^.

HIF-1α regulates mitochondrial biogenesis, and modulation of the nuclear-mitochondrial communication during aging depends on PGC-1α^79^. HIF-1α accumulates in the nucleus of both adult SIRT1 knockout mice and SIRT1-silenced primary myoblasts^85^. The increased HIF-1α levels observed during aging or in response to SIRT1 knockout activated the Mxi1 gene, which encodes a c-Myc transcriptional repressor. The Mxi1 gene product restricts the interaction between c-Myc and mitochondrial transcription factor A (TFAM) that is critical for replication, transcription, and maintenance of mitochondrial biogenesis^86^, further decreasing the TFAM promoter activity and suppressing mitochondrial biogenesis^79^.

SIRT1 mRNA transcription is also coupled with hypoxia during the aging process (Fig. 3). Feedback control mechanisms regulating levels of the SIRT1 transcript include the p53/forkhead box O-3a (FoxO3a)-SIRT1 pathway, FoxO1-SIRT1 pathway and HIF-1α/HIF-2α-SIRT1 pathway^73^. During acute hypoxia, HIF-1α and HIF-2α accumulate stably, interact with Hsp90, and directly bind to HREs in the SIRT1 promoter. During aging, PPAR1 activity is substantially enhanced, leading to NAD^+^ depletion and thereby inhibiting SIRT1 activity. In addition, the hypermethylated in cancer 1 protein (HIC1) and C-terminal-binding protein 1 (CtBP) complex associates with SIRT1 to repress SIRT1 transcription. A repressor complex, containing PPARγ or PPARβ and SIRT1, binds to specific sites in the SIRT1 promoter and directly suppresses transcription induced by exogenous stressors. The SIRT1 promoter region consists of a FoxO1 core binding repeat motif, and FoxO1-mediated SIRT1 transcription results in an increased expression of SIRT1. In response to DNA damage, acetylated E2F1 enhances the binding of FoxO1 to the SIRT1 promoter region, increasing SIRT1 transcription.

## Cross-talk between the SIRT1 and AMPK pathways during hypoxia and aging

AMPK is a heterotrimeric serine/threonine protein kinase composed of a catalytic α subunit and regulatory β and γ subunits. AMP binds to the γ subunit and activates AMPK by promoting Thr172 phosphorylation of the α subunit mediated by liver kinase B1 (LKB1)^87^. When the α subunit is phosphorylated on Ser485 by other upstream kinases, such as insulin-activated Akt, cAMP-dependent protein kinase (PKA), and RAS/MEK/ERK pathways, it is rendered inactive^88^. AMPK is activated by other serine/threonine kinases, such as Ca^2+^/calmodulin-dependent protein kinase β and transforming growth factor-β-activated kinase 1, and inhibited by several protein phosphatases, such as PP1, PP2A, and PP2C^89^.

AMPK is an important regulator of energy metabolism, stress resistance, and cellular proteostasis. AMPK also links energetics to longevity^90^. According to the results from several studies, the activation capacity of AMPK signaling decreases with aging, which impairs the maintenance of efficient cellular homeostasis and accelerates the aging process^89^. However, aging also enhances AMPK activation under certain conditions, suggesting that the aging process affects AMPK activation in a context-dependent manner.

AMPK activation extends the lifespan by reducing oxidative stress through an increase in thioredoxin levels, by reducing endoplasmic reticulum stress and inflammatory disorders, and by promoting autophagic clearance during the aging process^91^. In addition, active AMPK is involved in several aging-associated processes, such as cancer, insulin resistance, and osteoarthritis, characterized by defects in mitochondrial biogenesis^92^. Similar to SIRT1, activated AMPK modulates mitochondrial biogenesis via PGC-1α. Mitochondrial biogenesis is compromised in humans with osteoarthritis and aging chondrocytes, which is reversed by the activation of the AMPK-SIRT1-PGC1α pathway^92^.

**Fig. 4:**
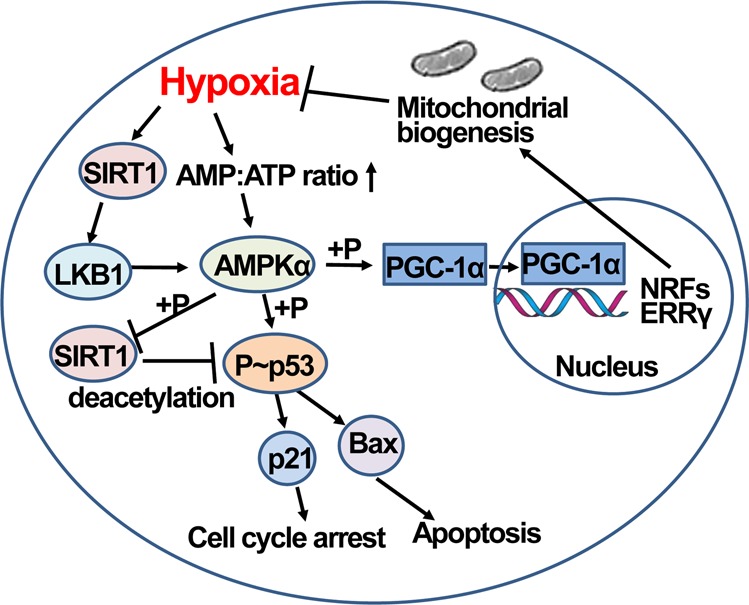
Hypoxia activates AMPK directly by increasing the AMP:ATP ratio or indirectly via SIRT1 activation. LKB1 is activated by SIRT1 and promotes the phosphorylation and activation of the catalytic α-subunit of AMPK. Activated AMPK further facilitates the phosphorylation and activation of p53, which induces the upregulation of p21 and Bax, resulting in cell cycle arrest and apoptosis. Moreover, AMPK induces the phosphorylation and inactivation of SIRT1, which induces the acetylation of p53. Activated AMPK increases the phosphorylation and activity of PGC-1α in the nucleus, triggering the transcription of mitochondrial genes, such as NRF1 and ERRγ, and enhancing mitochondrial biogenesis

Hypoxia is known to activate AMPK directly by increasing the AMP:ATP ratio or indirectly via SIRT1 activation (Fig. 4). LKB1 is activated by SIRT1 and promotes the phosphorylation and activation of the catalytic α-subunit of AMPK^93^. Activated AMPK directly phosphorylates and activates p53^94^, which induces the upregulation of cyclin-dependent protein kinase inhibitors and pro-apoptotic genes, such as p21 and Bax, respectively^95^, resulting in cell cycle arrest and apoptosis^96^. AMPK also induces the upregulation of p21 and Bax via the acetylation of p53 by AMPK-induced phosphorylation and inactivation of SIRT1^95^. In skeletal muscle, exercise-activated AMPK directly increases the phosphorylation of Thr177 and Ser538 and activity of PGC-1α in the nucleus, which triggers the transcription of mitochondrial genes, such as nuclear respiratory factors (NRFs) and estrogen-related receptor γ (ERRγ), and strengthens mitochondrial biogenesis (Fig. 4)^97^. Meanwhile, activated AMPK increases the expression of PGC-1α protein and subsequent formation of a complex with SIRT1-MyoD, which in turn binds to the promoter of PGC-1α, creating a positive feedback loop for PGC-1α expression^98^. In addition to SIRT1-mediated deacetylation of PGC-1α, acetylation of PGC-1α by the acetyltransferase general control nonderepressible 5 (GCN5) might be another mechanism controlling PGC-1α activity. Acetylation of PGC-1α decreases mitochondrial biogenesis^99^.

## Cross-talk between the AMPK and mTOR-ULK1 pathways during hypoxia and aging

Furthermore, relationships among AMPK, mTOR, ULK1 (the key regulatory protein required to initiate mammalian autophagy as the mammalian homologs of ATG1), and autophagy (the process by which cellular components are self-degraded) have been suggested under hypoxic conditions^100^. mTOR is a serine/threonine protein kinase that influences organismal lifespan in various species, including mammals^12,101^. mTOR exists in two distinct protein complexes, mTORC1 and mTORC2^102^. Rapamycin-sensitive mTORC1 regulates protein synthesis and cell growth through the phosphorylation of p70 ribosomal S6 kinase 1 (p70S6K1: Thr389) and initiation factor 4E-binding protein 1 (4E-BP1: Thr37/46)^103,104^. The PI3K/Akt pathway is one of classic upstream pathways of mTORC1 signaling, and the tuberous sclerosis protein 1 and 2 (TSC1/2) complex is a negative regulator of mTOR. Akt inactivates TSC2 via phosphorylation^105^, whereas AMPK phosphorylates and activates TSC2^106^.

**Fig. 5:**
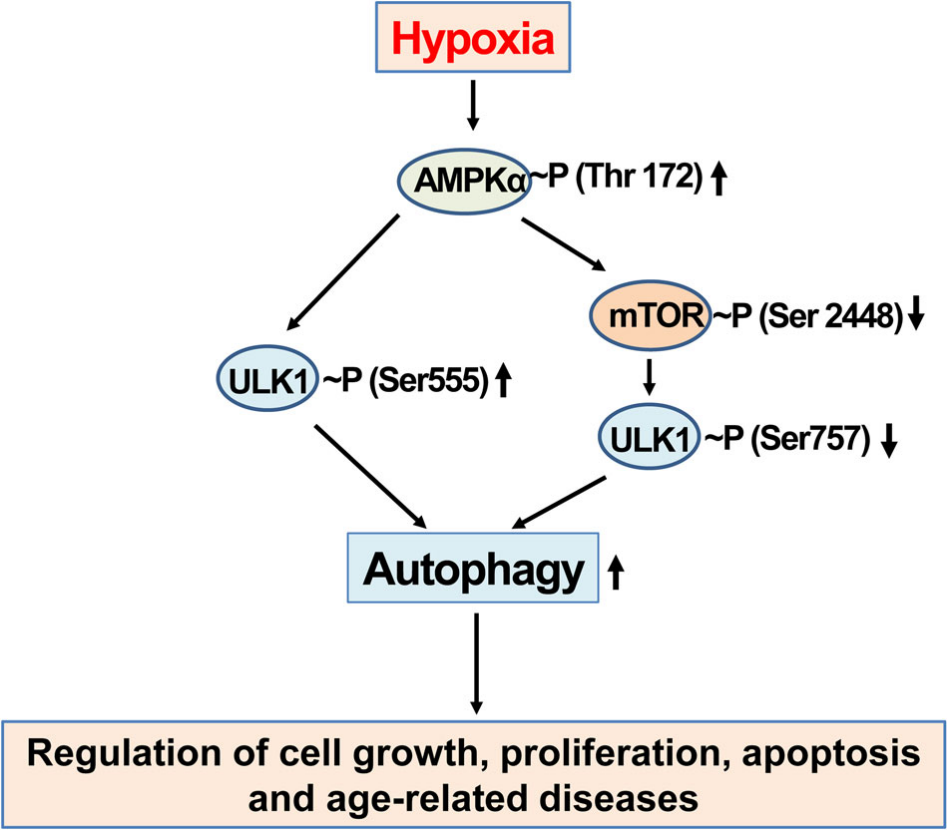
The relationship among AMPK, mTOR, ULK1 and autophagy under hypoxic conditions. During hypoxia, the levels of phosphorylated AMPK (Thr172) and phosphorylated ULK1 (Ser555) increase, and the levels of phosphorylated mTOR (Ser2448) and ULK1 (Ser757) decrease, resulting in an increase in autophagy which is responsible for regulating cell growth, proliferation, apoptosis and age-related diseases

Moreover, AMPK phosphorylates ULK1 either through direct or indirect pathways (Fig. 5). AMPK enhances autophagy by directly phosphorylating Ser555 in ULK1^107,108^. Meanwhile, AMPK inhibits the activity of mTOR to regulate cell growth and proliferation^106,109–111^ and mTOR inhibits autophagy by phosphorylating ULK1 at Ser757^107,112^. During hypoxia, the levels of mTOR, phosphorylated mTOR (Ser2448), and phosphorylated ULK1 (Ser757) are decreased, promoting autophagy, which is responsible for regulating cell growth, proliferation, apoptosis, and age-related diseases. Autophagy is initially increased as a compensatory response to hypoxia. Further enhancements in autophagy by chemical modulators may decrease cell growth and proliferation, increase autophagic cell death and apoptosis, and consequently regulate age-related diseases^100^.

Both the HIF and AMPK signaling pathways are evolutionarily conserved and play major roles in enabling survival during severe stress conditions, such as hypoxia and energy deficiency. The HIF-1-induced and AMPK-induced survival network includes protein kinases and other factors, such as SIRT1, mTOR, and ULK1, as well as several transcription factors, such as p53, FoxO, NFκB, and NRF2. HIF1 and AMPK activation increase not only the healthy lifespan but also the progression of tumorigenesis in a context-dependent manner^113^. HIF-1α increases angiogenesis by promoting the Src-dependent expression of VEGF in pancreatic and prostate carcinomas to help the cells adapt to harsh conditions^114^. Src activation leads to increased steady-state levels of HIF-1α and increased phosphorylation of STAT3. Both STAT3 and HIF-1α bind to the promoter of the VEGF gene, where they form a complex with the transcriptional coactivators, including p300/CBP and Ref-1/APE.

## Cross-talk between the AMPK and NFκB pathways during hypoxia and aging

**Fig. 6:**
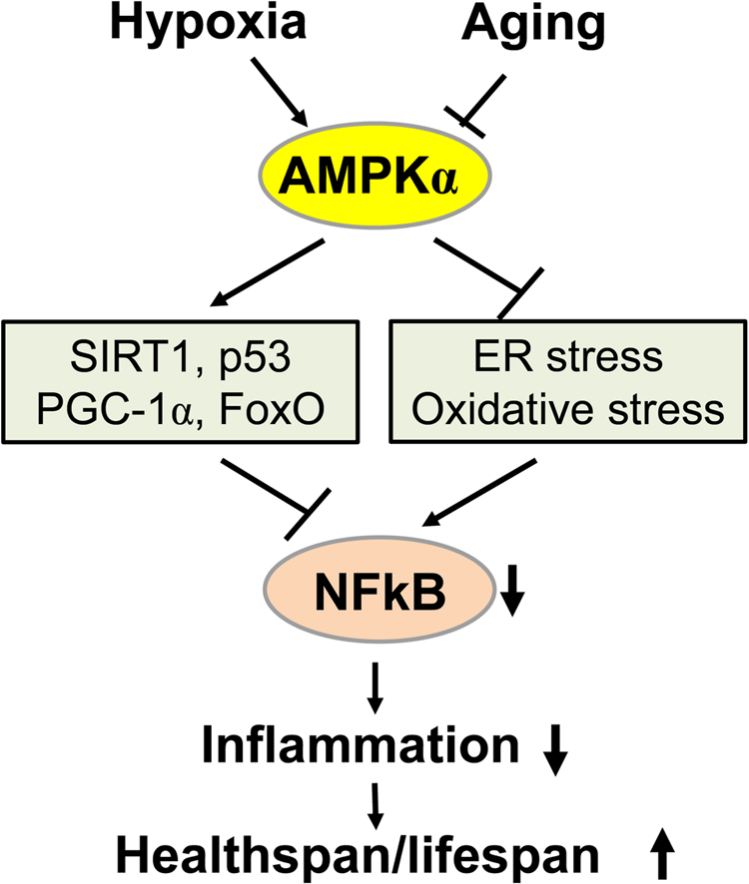
The AMPK pathway links hypoxia and aging to NFκB signaling and inflammation. Hypoxia activates AMPK. In contrast, aging appears to inhibit the expression and activity of AMPKα. Activated AMPK in turn stimulates SIRT1, p53, PGC-1α and FoxO, which inhibit NFκB signaling via different mechanisms. AMPK also inhibits the induction of ER and oxidative stress, which are responsible for activating NFκB signaling. NFκB is inhibited by the activated AMPK pathway, resulting in a reduction of inflammatory responses, which affect the healthspan and lifespan.^a^ Medizinische Klinik 4 and Translational Research Center, Universitätsklinikum Erlangen und Friedrich-Alexander-Universität (FAU) Erlangen-Nürnberg, Erlangen, Germany

Although the AMPK signaling pathway functions to improve energy metabolism and autophagy, it inhibits chronic stress-linked inflammation^115^. AMPK also inhibits endoplasmic reticulum and oxidative stress, which are involved in metabolic disorders and in the aging process. A low-level of chronic inflammation is observed during the aging process and the AMPK signaling pathway inhibits NFκB-induced inflammatory responses (Fig. 6)^115^. NFκB signaling is inhibited by the AMPK-dependent phosphorylation of several downstream targets, such as SIRT1, p53, PGC1α, and FoxO. AMPK-induced suppression of inflammatory responses has been found to augment the healthspan and lifespan.

The role of AMPK in cancer suppression has also been reviewed extensively^116^. Similar to aging, cancer also involves the induction of inflammation, and thus AMPK may play a role in preventing inflammatory responses in cancer cells. Therefore, the LKB1-AMPK signaling pathway was shown to be a powerful tumor suppressor pathway. AMPK agonists, such as metformin, hold promise as potent cancer drugs^116,117^. In agreement with this hypothesis, metformin inhibits the mTORC1 pathway via AMPK-dependent and AMPK-independent pathways, thereby inhibiting cancer cell growth and development^113,118^.

## Prenatal hypoxia and aging

**Fig. 7:**
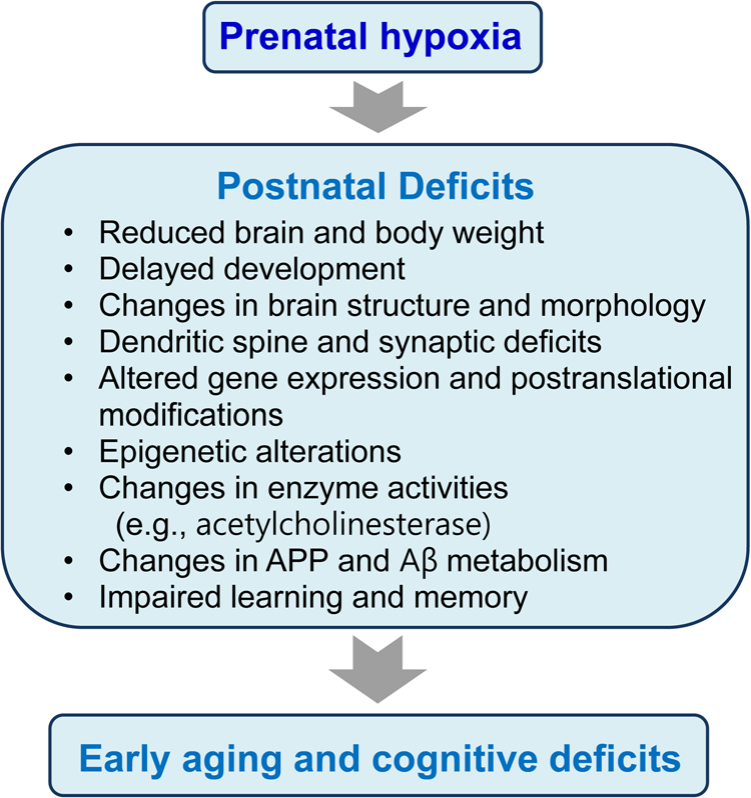
Postnatal deficits observed in animal models of prenatal hypoxia are responsible for early aging and cognitive deficits

Recently, experimental, epidemiological, and clinical studies have suggested critical roles for gestational (prenatal) factors in brain development and functions in the postnatal period. The prenatal factors increase the risks of various CNS disorders, such as autism, schizophrenia, Down syndrome, epilepsy, and depression, and predispose aging individuals to the development of diabetes and neurodegenerative disorders, including Parkinson’s and Alzheimer’s diseases^119^. Prenatal factors affecting fetal brain development include tobacco and alcohol, poor nutrition, maternal stress, placental deficits, hormones, toxins, and infections^120^. Since pregnancy complications, abnormal pregnancy and labor often lead to an insufficient oxygen supply to the fetus, prenatal hypoxia during the critical periods of brain development is one of the most important factors manifesting in early aging, mental retardation, and cognitive deficits at various postnatal stages of life^120^. An understanding of which type of changes are induced by prenatal hypoxia in the developing brain is important to prevent the development of CNS disorders later in life. These changes are outlined in Fig. 7.

In general, neuronal cells are more vulnerable to the effects of hypoxia than other types of cells^121^. Therefore, hypoxic insults to the brain have more significant consequences than in other organs and lead to severe pathologies^122^. Similarly, prenatal hypoxia may more severely affect the brain, resulting in a number of changes in its structural and functional properties^123^. Prenatal hypoxia reduces the action potentials and plasticity in the brain by preventing the formation of new contacts between cells and the propagation of neuronal signals, particularly in the cortex and hippocampus, which play important roles in learning and memory. Furthermore, prenatal hypoxia has a significant impact on the expression of a variety of genes, resulting in changes in the patterns of mRNA and protein expression and their post-translational modifications, including protein misfolding and clearance. Among these proteins, acetylcholinesterase, a key enzyme of the cholinergic system, and amyloid precursor protein (APP) are significantly affected by prenatal hypoxia^124,125^. Because these proteins have important roles in brain function, hypoxia-induced disruption of their expression and metabolism can result in early cognitive dysfunctions and the subsequent development of neurodegeneration. A decrease in the activity of NEP and other amyloid-β peptide (Aβ)-degrading enzymes was also observed after prenatal hypoxia, resulting in an Aβ clearance deficit, accumulation of its toxic species, and subsequent neuronal cell death and neurodegeneration (Fig. 7).

In addition, prenatal hypoxia has been shown to impair the response of the developing organism to hypoxia in adulthood by inducing alterations in catecholaminergic components of the chemoafferent pathway contributing to impaired postnatal respiratory behavior^126^. Hypoxia-induced down-regulation of NEP might contribute to these alterations^127^. Adult rats exposed to intermittent hypoxia in the neonatal period exhibit an augmented carotid body and adrenal chromaffin cell response to hypoxia and irregular breathing, which are associated with increased oxidative stress^128^. The application of various approaches to restore expression of neuronal genes that are disrupted by prenatal hypoxia during postnatal development opens an avenue for the therapeutic compensation of cognitive dysfunctions and prevention of Aβ accumulation in the aging brain and the model of prenatal hypoxia in rodents, which can be used as a reliable tool to assess their efficacy.

## OSA-induced intermittent hypoxia and aging

OSA is a sleep-related breathing disorder characterized by repeated episodes of complete (apnea) or partial (hypoapnea) obstruction of the upper airway. OSA causes cyclical hypoxemia-reoxygenation and stimulates chemoreceptors, resulting in over-activation of SNA, bursts in systemic blood and arterial pulmonary pressures^6^, and concomitant cellular chronic intermittent hypoxia^129^. Long-term consequences of OSA or chronic intermittent hypoxia include molecular and cellular impairments that contribute to the accelerated progression and severity of many diseases, including cardiovascular diseases^130,131^, metabolic diseases^132^, neurological disorders^131,133,134^, cancer and aging^135^. The pathophysiological consequences and clinical manifestations of OSA or chronic intermittent hypoxia are summarized in Table 3.

**Table 3:** The pathophysiological consequences and clinical manifestations associated with obstructive sleep apnea or chronic intermittent hypoxia

1. Cardiovascular diseases	
Pathophysiologic consequences	Clinical manifestations
• Hypoxemia, cyclic hypercapnia and hypocapnia • Nocturnal and diurnal hypertension • Alterations in coronary blood flow • Increased sympathetic activity • Increased oxidative stress • Increased inflammatory response (elevated levels of C-reactive protein, NFκB, TNF-α, IL-8, IL-6, and EPO) • Increased levels of adhesion molecules (ICAM-1) • Endothelial dysfunction (increased big ET-1 or ET-1 level, reduced nitric oxide level, and activation of the renin-angiotensin system) • Increased coagulation (fibrinogen) • Increased platelet aggregation • Increased blood viscosity	• Systemic hypertension • Congestive heart failure (systolic and diastolic dysfunction) • Coronary artery disease • Arrhythmias • Pulmonary hypertension • Stroke • Atherosclerosis
2. Metabolic diseases	
Pathophysiological consequences • Activation of the sympathetic system • Increased release of adipocyte-derived inflammatory mediators (IL-6, TNF-α, and leptin) • Activation of hypothalamus-pituitary-adrenal axis (increased cortisol levels)	Clinical manifestations • Glucose intolerance and insulin resistance • Type 2 diabetes mellitus • Metabolic syndrome
3. Neurological disorders	
Pathophysiological consequences	Clinical manifestations
• Increased activity of sympathetic neuron • Increased oxidative stress • Increased neuronal apoptosis within the cortex and CA1 region of the hippocampus • Increased expression of the COX-2 protein and gene	• Depression • Insomnia and/or excessive daytime somnolence • Cognitive and neurobehavioral abnormalities: impairments in concentration, perception, memory, performance and learning • Attention deficit hyperactive disorder (ADHD) • Headaches
4. Cancer and aging	
Pathophysiological consequences	Clinical manifestations
• Increased oxidative stress • Molecular and cellular impairments • Accelerated cellular aging	• Tumors • Cancer
5. Other functional defects	
Pathophysiological consequences	Clinical manifestations
• Reduced upper airway muscle endurance and impaired pharyngeal dilator • EMG responses to physiological stimulation	• Gastroesophageal reflux • Genitourinary: nocturia and sexual dysfunction • Toxemia (preeclampsia/eclampsia) during pregnancy

**Fig. 8:**
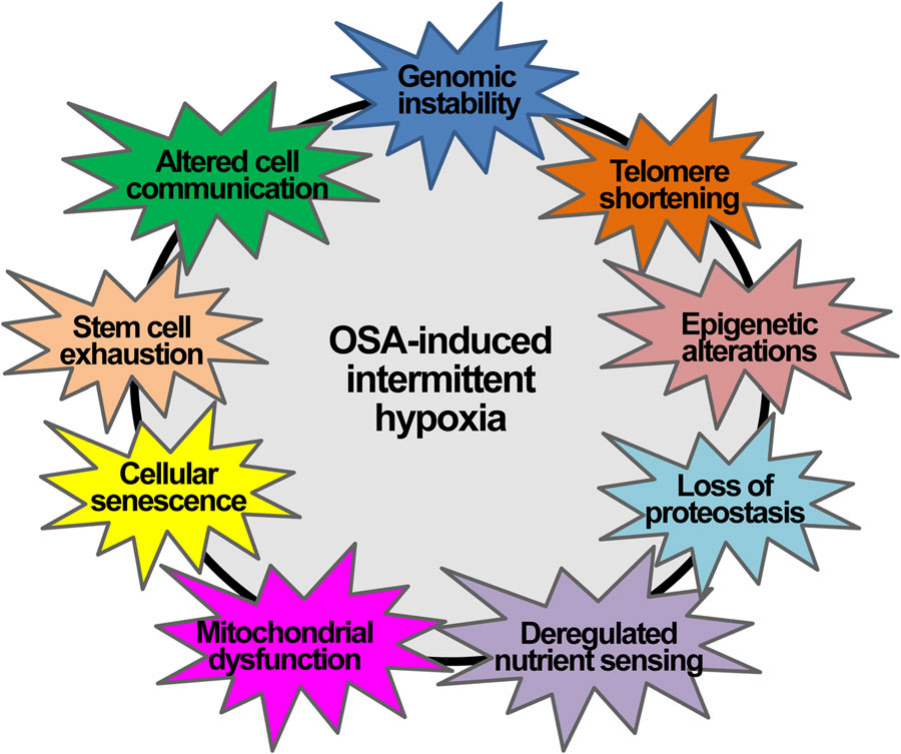
The hypothetical impact of OSA-induced intermittent hypoxia on aging and aging-related diseases. OSA-associated intermittent hypoxia may promote the induction of the nine hallmarks of aging: genomic instability, telomere shortening, epigenetic alterations, loss of proteostasis, deregulated nutrient sensing, mitochondrial dysfunction, cellular senescence, stem cell exhaustion, and altered intercellular communication. These changes accelerate aging and aging-related diseases, such as cancer

Previously, hypoxia was shown to potentially contribute to cellular aging and the functional decline during the aging process. OSA-associated intermittent hypoxia may also accelerate cellular aging and age-related diseases by prompting the induction of nine hallmarks of aging: genomic instability, telomere shortening, epigenetic alterations, loss of proteostasis, deregulated nutrient sensing, mitochondrial dysfunction, cellular senescence, stem cell exhaustion, and altered cellular communication (Fig. 8)^136^.

Because aging is one of the risk factors for cancers and most hallmarks of aging have been shown to accelerate tumorigenesis, resulting in cancers, OSA is also postulated influence tumorigenesis and carcinogenesis^135,137^. Tumor physiology, animal and epidemiological human studies reveal a strong relationship between OSA and cancer. However, because current data relating OSA to neoplastic diseases remain scarce, more research on the impact of OSA on cancer-related aspects are needed.

## Summary

Due to its crucial role in metabolism and survival, hypoxia has attracted the interest of many researchers. Hypoxia-inducible factors (HIFs) form an efficient and rapid oxygen sensing system, and effectively control the hypoxic responses, which induce the expression of several adaptive genes to increase the oxygen supply and support anaerobic ATP generation in eukaryotic cells. Aging-associated cumulative damages and degenerative changes lead to dysfunction and failure at the cellular and tissue levels, ultimately resulting in disorders of whole body function. Hypoxia potentially contributes to functional decline during the aging process. We must improve our understanding of the molecular and cellular responses to hypoxia to reduce the hypoxia-induced damage and senescence induction.

HIF pathways cross-talk with sirtuins, AMPK, and mTORC1-ULK1 pathways, which are involved in the mechanisms regulating inflammation, mitochondrial biogenesis, cellular senescence, and organismal aging. The putative molecular mechanisms underlying the effects of hypoxia, including HIF-1α, AMPK, sirtuins, and mTORC1, are discussed in this manuscript. The levels of these proteins are controlled by both hypoxia and aging; the level of SIRT1 is decreased at both transcriptional and posttranscriptional levels during aging, which attenuates mitochondrial biogenesis and causes aging-related diseases. The activity of SIRT1 is also regulated by an aging-associated reduction of the nuclear energy state and NAD^+^ levels, resulting in diminished levels of the pVHL protein and stabilization of HIF-1α. Conversely, overexpression of SIRT1 promotes mitochondrial biogenesis and the subsequent activation of HIF-1α and PGC-1α. The upregulation of SIRT1 may prevent premature cellular senescence and the pathogenesis of many aging-related chronic diseases.

AMPK is an important regulator of energy metabolism, stress resistance, and cellular proteostasis. AMPK also links energetics to longevity and its activation extends the lifespan. The activation capacity of AMPK signaling decreases with aging, which impairs the maintenance of efficient cellular homeostasis and accelerates the aging process. Similar to SIRT1, activated AMPK modulates mitochondrial biogenesis via PGC-1α. A deficiency in mitochondrial biogenesis is reversed by the activation of the AMPK-SIRT1-PGC-1α pathway. Hypoxia activates AMPK directly by increasing the AMP:ATP ratio or indirectly via SIRT1 activation. LKB1 is the protein activated by SIRT1, which promotes the phosphorylation and activation of the catalytic α-subunit of AMPK.

Hypoxia contributes to cellular aging and the functional decline observed during the aging process. Prenatal hypoxia during the critical periods of brain development could manifest as early aging, mental retardation, and cognitive deficits in postnatal life. OSA-associated intermittent hypoxia may also accelerate cellular aging and aging-related diseases by prompting the induction of the nine hallmarks of aging: genomic instability, telomere shortening, epigenetic alterations, loss of proteostasis, deregulated nutrient sensing, mitochondrial dysfunction, cellular senescence, stem cell exhaustion, and altered cellular communication.
